# Deploying external bandwidth guaranteed media server clusters for real-time live streaming in media cloud

**DOI:** 10.1371/journal.pone.0214809

**Published:** 2019-04-03

**Authors:** Weizhan Zhang, Zhichao He, Biao Du, Minnan Luo, Qinghua Zheng

**Affiliations:** MOEKLINNS Lab, Department of Computer Science and Technology, Xi’an Jiaotong University, Xi’an, China; Pablo de Olavide University, SPAIN

## Abstract

The cloud-based media streaming service is a promising paradigm for multimedia applications. It is attractive to media streaming service providers, who wish to deploy their media server clusters in a media cloud at reduced cost. Since the real-time live streaming service is both a bandwidth-intensive and quality-sensitive application, how to optimize the internal bandwidth utilization of a data center network (DCN) as well as guarantee the external bandwidth of the real-time live streaming application, is a key issue of deploying virtual machine (VM)-hosted media server cluster in a media cloud. Therefore, in this study, we propose an external-bandwidth-guaranteed media server cluster deployment scheme in media cloud. The approach simultaneously considers the outside bandwidth requirement of a tree-based media server cluster for live streaming and the intra-bandwidth consumption of a DCN. The proposed scheme models the optimal problem as a new terminal-Steiner-tree-like problem and provides an approximate algorithm for placing the media servers. Our evaluation results show that the proposed scheme guarantees the external bandwidth requirement of a real-time live streaming application, at the same time, greatly reduces the intra-bandwidth utilization of a media cloud with different DCN structures.

## Introduction

Due to the centralized management of elastic resources, the cloud can provide scalable data storage, computation, and networking services at a reduced cost [1]. Thus, media streaming service providers are enticed to deploy their applications in the cloud. Recently, the media cloud came into existence [2]. Some researchers have already studied how the media cloud can be efficiently used for cloud-based media streaming applications [2, 3, 4, 5, 6, 7, 8, 9, 10, 11, 12, 13, 14, 15, 16]. Because these media streaming applications are resource-intensive, how to maximize resource utilization is still a major challenge for cloud-based media streaming service providers.

Virtualization technology provides an effective means to improve the resource utilization of the cloud. Many recent studies have concentrated on virtual machine placement and migration in the cloud. In these studies, the deployment issue of the virtual machine (VM) server is often described as a multidimensional vector packing problem. Optimization objectives often focus on resource utilization, networking overhead, energy efficiency, and migration times. The solutions include dynamic deployment or static placement, and their goals are mainly achieved using the heuristic greedy algorithm to obtain an approximate optimal solution [17]. Among them, several studies have taken the internal bandwidth resources of a data center network (DCN) into account [18, 19, 20, 21, 22, 23, 24, 25]. These traffic-aware solutions optimize the resource utilization of intra-bandwidth in a DCN, which is the critical bottleneck resource in a media cloud [26]. However, these VM management studies mainly took the infrastructure of the cloud into account, lacking consideration of the quality requirement of media streaming applications. Therefore, they cannot be adopted directly for many media streaming applications.

This study focuses on deploying real-time live streaming services in a media cloud. The media server cluster of a real-time live streaming service has special properties. The popular HTTP streaming or HTTP adaptive streaming architecture cannot be adopted under this context because of the pull-based nature of HTTP. Real-time live streaming services in media cloud, such as time-sensitive video conferencing and virtual classrooms, usually employ a tree-based media server cluster in data center. They usually let the root VM push the video to other VMs to provide real-time, large-scale streaming services. In this situation, the leaf VM nodes in the tree truly provide the real-time live streaming services for clients simultaneously. In this manner, the service provider can isolate server internal traffic and external video streaming for user application, to enhance the overall service quality. Therefore, the external bandwidth of the leaf VM nodes, connecting the clients outside the data center, should be guaranteed to ensure the quality of the large-scale services. For example, real-time live streaming applications based on web real-time communication (WebRTC) [27] may need a tree-based multipoint controller (MCU) cluster to enlarge the one-to-many real-time live streaming service. This research is motivated by the case of deploying a tree-based MCU media server cluster in a media cloud for a virtual classroom system. Therefore, to fully utilize the resources of the cloud as well as to provide high-quality, real-time live streaming services, the deployment strategy used for a media server cluster needs to consider both the inside and outside bandwidth resources of the data center. In this manner, the service requirement of media streaming can be fully known and guaranteed, and the efficient internal bandwidth utilization of the media cloud services can also be achieved.

**Fig 1:**
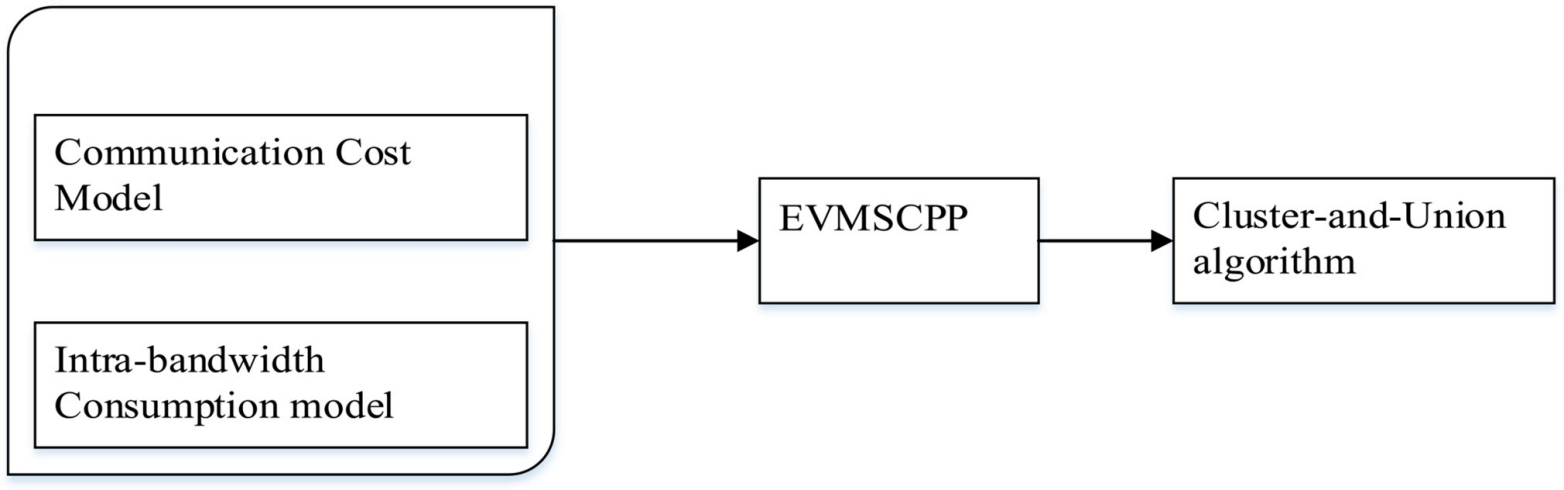
Model of the core idea.

In this study, we propose a VM-hosted media server cluster deployment scheme for external-bandwidth-guaranteed, real-time live streaming services. Firstly, the scheme proposes a mathematical model to illustrate the services deployment problem and demonstrates the difficulty. Then, a greedy media server placement algorithm is introduced to optimize the internal bandwidth utilization of a DCN under the application constraints of real-time live streaming services. Fig 1 shows the schematic description of the whole method. The approach contributes the following new ideas.

- First, the approach simultaneously considers the outside bandwidth requirement of a tree-based media server cluster for live streaming and the intra-bandwidth consumption of a DCN. The approach models this specific application requirement as a newly defined terminal-Steiner-tree-like problem, extending the target and domain of the traditional VM management algorithms.
- Second, the approach designs an approximation algorithm called cluster-and-union to deploy the media streaming cluster. The algorithm ensures that the number of leaf nodes of the tree-based media sever cluster meets the bandwidth requirement of the real-time live streaming service, and at the same time, obtains an approximate optimal minimum tree solution to minimize the intra-bandwidth consumption of the DCN.

The remainder of this paper is organized as follows. We summarize the related works in Section 2. We give the problem formulation, establish the mathematical model, and introduce the approximation algorithm solution in Section 3, and then we evaluate the performance of our proposal via a simulation in Section 4. Finally, we conclude the paper and discuss potential future works in Section 5.

## Related works

### Media cloud computing

Recently, taking advantage of the centralized management of elastic resources in cloud computing, media cloud computing has come into existence [2]. Research on media cloud computing mainly focuses on resource management of the media cloud. Researchers have tried to enhance media streaming services by optimizing the resource allocation of the media cloud to fully utilize the elastic and on-demand nature of cloud resource provision. Specifically, the authors of [28] proposed a novel SDN-based game-aware network management method for cloud gaming. The scheme assigns game servers and selects the communication path by considering the types of games, sever loads, and path delays. GamingAnywhere [29] also concentrated on cloud gaming and provided an open source system. DRES [6] derived an optimal redirection policy under a cloud-centric media network, which reallocates user requests to multiple VM hosts to scale the service capacities. The research in [30] provided a generalized framework for computing the resources required to support multiple services via the utilization of an earliest deadline first strategy. DCC-VOD [7] presented a distributed video-on-demand system design based on cloud computing, focusing on the implementation of load balancing among servers. OPT-ORS [8] investigated the tradeoff between the cost incurred by VM instance procurement and the achieved QoE of end users under commercial pricing models. DREAM [9] proposed a distributed heuristic algorithm, aiming to solve the resource reservation and scheduling problem of configuring the cloud utility to meet SLAs for VoD applications at a modest cost. CALMS [13] adaptively leased and adjusted cloud server resources in a granularity to accommodate the temporal and spatial dynamics of demands from live streaming users. The research [15] introduced a segment-based storage and transcoding trade-off strategy for multi-version VoD systems in the cloud. In addition, some media cloud computing studies also examined the load balancing problem among servers for multimedia computing, such as for image enhancement or video transcoding, to efficiently utilize the parallel computing power of the cloud [16, 31].

In summary, some studies have focused on providing media streaming services based on the media cloud. Among them, some have already considered the resource allocation among different VM-hosted servers. However, as of now, few efforts have been devoted to exploring in detail how the server cluster of real-time live streaming can be deployed in a DCN.

### Traffic-aware VM management in the cloud

Virtualization technology provides an effective means to optimize the resource utilization of an elastic cloud, which has recently been studied intensively. Most related VM management approaches can be roughly categorized as either VM placement or VM migration schemes.

VM placement or migration studies address the problem of how to place or replace VMs in a physical machine according to the specific optimization needs. In these studies, the management issue of the VM server is often described as a multidimensional vector packing problem with different optimization objectives. Among them, some researchers have already paid attention to the internal bandwidth consumption of a DCN. The research presented in [18] proposed a traffic-aware VM placement algorithm to improve network scalability. The study optimized the placement of VMs on host machines, by which traffic patterns among VMs can be aligned with the communication distance between hosts. The research [19] proposed an online joint VM placement and routing algorithm to minimize the traffic costs of a DCN, which employed VM migration to combine the VM placement and the data routing among VMs. The authors of [20] focused on maximizing the benefit of the overall communication sent by the VMs to a single designated point in the data center. Shadow [21] proposed a combined VM routing and VM placement algorithm, named the Shadow scheme, which minimizes the maximum appropriately defined data center utilization. The research presented in [22] focused on the optimized placement of VMs to minimize the cost by balancing DCN traffic and the utilization of physical machines. The authors of [23] analyzed how much bandwidth is required to guarantee the total migration time and downtime of live VM migrations and gave the bandwidth value required to satisfy the performance metrics of live VM migrations. Vmbuddies [24] designed and implemented a coordination system for correlated VM migrations, which is one of the earliest researches to solve the correlated migration problem of multi-tier application in cloud environments. The study migrates tightly-coupled VMs simultaneously, and minimizes the migration costs of multi-tier applications. Our precious work [25] also proposed a VM collaborative migration scheme. It targets at media cloud shared by concurrent media applications, and provides a generalized solution to solve the problem of VM migration in media cloud.

In summary, these studies focused on multiobjective constrained VM placement or migration. Some studies have already taken the intra-traffic of a DCN as the constraint, but the relevant studies did not consider the tree-based server clusters or bandwidth requirement of real-time live streaming.

## Materials and methods

### Communication cost model of DCN

The internal bandwidth of DCNs has increasingly become a bottleneck restricting the utilization of DCNs [1]. The VM-hosted media server cluster placement scheme should be fully aware of the DCN structure since media applications are bandwidth intensive. In recent years, researchers have carried out extensive studies on DCN construction [32]. According to different construction rules and interconnected technologies, DCNs can be roughly categorized as either switch-based DCNs, server-based DCNs, module-based DCNs, or random-based DCNs.

Although the topologies and the interconnection rules of DCNs vary, their communication cost model can be uniformly defined as a weighted connected graph. Suppose that *G* = (*V*, *E*, *w*) is a simple undirected weighted complete graph with *n* vertices and *n*(*n* − 1)/2 edges. The vertex set *V* in *G* indicates the deployment point (DP) set in the DCN in which a physical machine is placed for hosting VMs. *E* is defined as the set of edges connecting two DPs. *w* denotes the weighting function of *E*. The value of *w*(*u*, *v*), *u*, *v* ∈ *V*, indicates the communication cost between the DPs *u* and *v*. It denotes the minimum number of links by which data is transferred. In addition, the weighting function *w* satisfies the following triangle inequality: *w*(*u*, *v*) < *w*(*u*, *a*) + *w*(*a*, *v*), *a*, *u*, *v* ∈ *V*.

In this manner, all of the communication costs between two arbitrary DPs composes an intra-communication cost matrix, which is denoted by *C* = {*c*_*ij*_}_*n*×*n*_, where *c*_*ij*_ = *w*(*v*_*i*_, *v*_*j*_). Taking a traditional three-tier tree-based DCN as an example, the communication cost between DP *v*_*i*_ and DP *v*_*j*_ is 2 when they are connected via the same access switch and is 4 when they are connected to two different access switches belonging to the same aggregation switch. If they belong to different aggregation switches, the communication cost between DP *v*_*i*_ and DP *v*_*j*_ is 6.

### Intra-bandwidth consumption model of DCN

For a VM-hosted cluster-based application, the traffic pattern among VMs can also be described as a weighted connected graph *G*′ = (*V*′, *E*′, *w*′).

A vertex $v_{i}^{{}^{\prime }}$, $v_{i}^{{}^{\prime }}\in V^{{}^{\prime }}$, indicates a VM in the cluster, and an edge $\left(v_{i}^{{}^{\prime }},v_{j}^{{}^{\prime }}\right)$ indicates traffic flow between $v_{i}^{{}^{\prime }}$ and $v_{j}^{{}^{\prime }}$. $w^{{}^{\prime }}\left(v_{i}^{{}^{\prime }},v_{j}^{{}^{\prime }}\right)$ shows the average traffic rate between $v_{i}^{{}^{\prime }}$ and $v_{j}^{{}^{\prime }}$; if $\left(v_{i}^{{}^{\prime }},v_{j}^{{}^{\prime }}\right)\notin E^{{}^{\prime }}$, $w^{{}^{\prime }}\left(v_{i}^{{}^{\prime }},v_{j}^{{}^{\prime }}\right)=0$. Thus, the traffic rate matrix for the VM-based cluster application can be denoted by *D* = {*d*_*ij*_}_*m*×*m*_, where $d_{ij}=w^{{}^{\prime }}\left(v_{i}^{{}^{\prime }},v_{j}^{{}^{\prime }}\right)$. Suppose that function *ψ* maps the VM to the DP. For instance, if the VM $v_{i}^{{}^{\prime }}$ is deployed to the DP *v*_*j*_, then $\psi \left(v_{i}^{{}^{\prime }}\right)=v_{j}$. Thus, it is easy to quantify the intra-bandwidth consumption caused by deploying the logic structure-known VM-based cluster application in a DCN. This consumption can be modeled using the following formula:

$$\begin{array}{c} \chi \left(G,G^{{}^{\prime }},\psi \right)=\sum_{\left(v_{i}^{{}^{\prime }},v_{j}^{{}^{\prime }}\right)\in E^{{}^{\prime }}}w^{{}^{\prime }}\left(v_{i}^{{}^{\prime }},v_{j}^{{}^{\prime }}\right)*w\left(\psi \left(v_{i}^{{}^{\prime }}\right),\psi \left(v_{j}^{{}^{\prime }}\right)\right) \end{array}$$ (1)

### External-bandwidth-guaranteed, VM-hosted media server cluster placement problem (EVMSCPP)

In this section, we define and formulate the external-bandwidth-guaranteed, VM-hosted media server cluster placement problem (EVMSCPP) based on the communication cost model and the intra-bandwidth consumption model of a DCN. We apply it to a terminal-Steiner-tree-like problem and then analyze its complexity.

The proposal focuses on a VM-hosted, cluster-based, real-time live streaming application such as large-scale live video broadcasting or a virtual classroom. Such applications organize the VM cluster into a tree-like topology structure. Only the VMs, functioning as leaf nodes in the tree, provide real-time live streaming services for clients simultaneously, as they are allocated sufficient external bandwidth resources. Suppose that they receive a copy of media data produced by the root VM at the rate of *d* and that each VM leaf node in the cluster is allocated the same output bandwidth resource *b*. In this manner, if the guaranteed external bandwidth requirement of the media streaming application is *B*, we should construct a logical tree topology cluster with *k* = ⌈*B*/*b*⌉ leaf nodes and determine a way to deploy the tree-like VM cluster to physical hosts in the DCN. To optimize the intra-bandwidth consumption of the DCN caused by media server cluster deployment, we can define the EVMSCPP as a problem that involves constructing a tree *T* that represents the topology of the VM-host media server cluster, with a constraint on the number of leaf nodes, and finding the VM placement method, denoted as *ψ*, used to minimize the following objective function:

$$\begin{array}{c} \begin{array}{c} \chi \left(G,T,\psi \right)=d\sum_{(v_{i},v_{j})\in T}w_{G}\left(\psi \left(v_{i}\right),\psi \left(v_{j}\right)\right)\\ \;s.t.\\ leaf(T)=k\in (0,|V(G)|)\wedge |V(T)|\leq |V(G)| \end{array} \end{array}$$ (2)

Note that *leaf*(*T*) stands for the number of leaf nodes in tree *T*, *d* = *w*_*T*_(*e*) is a constant and *ψ*: *V*(*T*) → *V*(*G*) is an injective function that reflects the mapping relation between the VM and DP.

In formula 2, when *G*, *k*, and *d* are given, minimizing the intra-bandwidth consumption of the DCN *χ* is equivalent to minimizing the simplified intra-bandwidth consumption of the DCN *φ*(*G*, *T*′), as shown in the following objective function:

$$\begin{array}{c} \begin{array}{c} \min_{T^{\prime }\in \prod }\left\{\varphi \left(G,T^{{}^{\prime }}\right)=\sum_{\left(v_{i},v_{j}\right)\in T^{{}^{\prime }}}w_{G}\left(v_{i},v_{j}\right)\right\}\\ \;s.t.\\ \prod =\{T^{{}^{\prime }}|V\left(T^{{}^{\prime }}\right)\subset V\left(G\right)\wedge E\left(T^{{}^{\prime }}\right)\subset E\left(G\right)\wedge leaf\left(T^{{}^{\prime }}\right)=k\} \end{array} \end{array}$$ (3)

That is, the EVMSCPP above (formula 3) tries to find a subtree *T*′ with *k* leaves whose weight is minimum in a weighted complete graph *G*. The simplified intra-bandwidth consumption *φ*(*G*, *T*′) represents the sum of the communication costs among DPs caused by *T*′. It is similar to the terminal Steiner tree (TST) problem in mathematics. The TST problem is defined as follows [33]. Let *G* = (*V*, *E*, *w*) be a positive edge-weighted complete graph with vertex set *V*, edge set *E*, edge weighting function *w* satisfying the triangle inequality, and target vertex set *S* ⊂ *V*. The terminal Steiner tree problem involves finding a minimum weighted subtree denoted by T* in *G* that interconnects all target vertices such that every target vertex in *S* appears as a leaf vertex in the subtree T*. From the definition of the TST problem, the set of leaf nodes S in the TST problem is given. However, in our EVMSCPP, only the number of leaf nodes *k* is known. The elements of the leaf nodes can be selected from the entire vertex set *V* and are unknown in advance. Thus, the terminal Steiner tree problem is a special case of the EVMSCPP. The TST problem has been demonstrated to be NP-hard and MAX SNP-hard (APX-hard) [2]. Thus, the EVMSCPP is also NP-hard and has a nondeterministic polynomial time solution. In the next section, we will introduce the approximation algorithm solution of the EVMSCPP in detail.

### Approximation solution of the EVMSCPP

There have been some studies on the approximate solution of the TST problem. For example, GH Lin put forward a three-step TST approximation algorithm [33]. In the EVMSCPP, since the set of leaf nodes is nondeterministic, the traditional approximation algorithms of the TST problem cannot be directly adopted to solve our problem. We need to choose the leaf node set from *G* in advance before we use the corresponding approximate TST solutions. We can give an analysis of the approximation ratio for employing the TST approximate solutions to solve our EVMSCPP problem.

***Lemma*** Suppose that there is an approximation algorithm for the TST problem with approximation ratio *ρ*; then, there is also a corresponding approximation algorithm for the EVMSCPP with approximation ratio *ρ*.

***Proof*** Let *T*′ denote an optimal tree in *G* such that *leaf*(*T*′) = *k*. Let *S*_*i*_, $i\in \{1,2,...,C_{\left|G\right|}^{k}\}$, denote a vertices set in *G*, and |*S*_*i*_| = *k*, $\forall i\in \{1,2,...,C_{\left|G\right|}^{k}\}$. Let *T*^*i*^ denote the TST tree for target vertices |*S*_*i*_| in *G* obtained using one TST approximation algorithm. Let T* denote the approximate EVMSCPP tree obtained by employing the TST approximation algorithm $C_{\left|G\right|}^{k}$ times. That is, T* is the minimum tree selected from *T*^*i*^, $i\in \{1,2,...,C_{\left|G\right|}^{k}\}$, by employing the TST approximation algorithm $C_{\left|G\right|}^{k}$ times. By definition,

$$\begin{array}{c} \begin{array}{c} w_{G}\left(T^{*}\right)=min\left(w_{G}\left(T^{i}\right),i=1,2,...,C_{\left|G\right|}^{k}\right)\\ \leq w_{G}\left(T^{i}\right),\forall i\in \left\{1,2,...,C_{\left|G\right|}^{k}\right\}\;\;\;\\ \leq \rho w_{G}\left(T^{\prime }\right)\;\;\;\;\;\;\;\;\;\;\;\;\;\;\;\;\;\;\;\;\;\;\;\;\;\; \end{array} \end{array}$$ (4)

where *ρ* is the approximation ratio of the adopted TST approximation algorithm. This proves the lemma.

The proof indicates that approximation solutions of the EVMSCPP can always be obtained with the same approximation ratio by employing the approximation solutions of the TST problem. However, this means that the complexity of the EVMSCPP approximation algorithm is $C_{\left|G\right|}^{k}$ times that of the corresponding TST approximation algorithm. Therefore, we further propose another approximation algorithm called “cluster-and-union” in this study, which leverages the network topology feature of data centers. The approximation ratio of the cluster-and-union algorithm is related to the different DCN structures for specific applications, but the complexity of the proposed cluster-and-union approximation solution is reduced to O(n). The algorithm is as follows. As mentioned above, the EVMSCPP is NP-hard. However, if *k* = |*G*| − 1, then it is easy to find a subtree *T* whose vertex set covers all the vertices in *G* and in which only one vertex (i.e., the root) is a non-leaf node, with the other vertices functioning as leaves. Thus, the main idea in solving this problem is to reduce the EVMSCPP to several, small special cases and then to unify the subtrees constructed in those special cases. The cluster-and-union algorithm is mainly composed of three steps.

**Fig 2:**
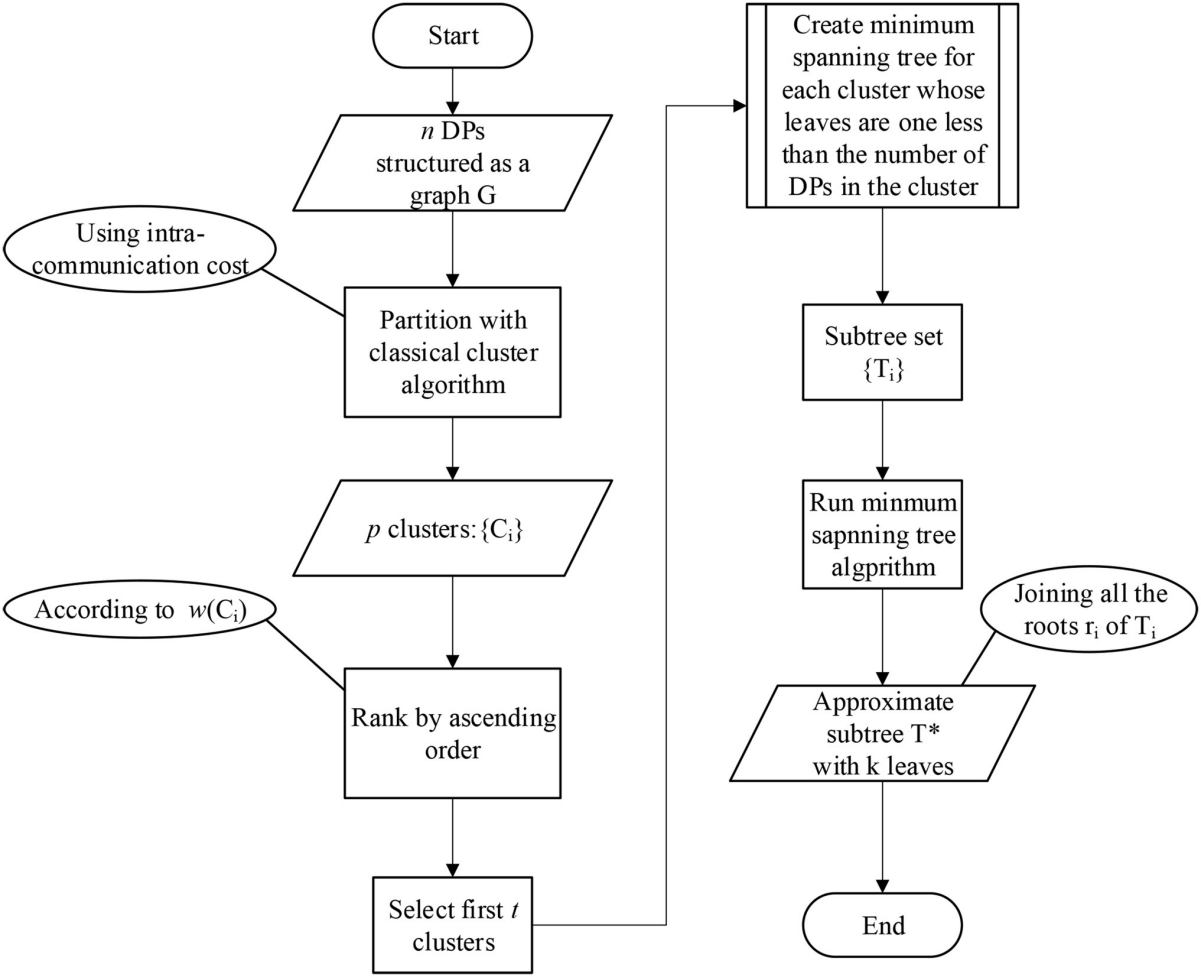
Workflow of Cluster-and-Union algorithm.

First, we partition the *n* DPs into *p* clusters by using the intra-communication cost among DPs and then sort the set of clusters according to the simplified intra-bandwidth consumption value. We run the classical clustering algorithms based on the intra-communication cost matrix that satisfies the triangle inequality to partition the DPs into several clusters. Taking the three-tier architecture network of DCNs as an example, the clustering characteristic of DPs is obvious, and DPs connected to the same access switch can be gathered as a cluster. Thus, the number of clusters *p* can be set to the number of access switches in the DCN. Second, we select the first *t* clusters, the total simplified intra-bandwidth consumption costs of which are the top *t* costs when ranked in ascending order, and make sure that the sum of the numbers of leaf nodes of all subtrees created from the clusters is not less than *k*. For each cluster, we create a subtree whose number of leaves is one less than the number of DPs in each cluster and whose weight is minimum. Notice that the minimum span tree will be the same as the EVMSCPP tree in this special case. Finally, after obtaining *t* subtrees with a total of *k* leaves and *t* roots via the previous steps, we unify the *t* subtrees to construct the final approximate subtree by joining the *t* roots after running the classical minimum spanning tree algorithm. The pseudocode for the algorithm is described below. Fig 2 shows the workflow of the algorithm.

**Algorithm 1** Cluster-and-Union

INPUT:

*G* (Simple undirected weighted complete graph).

*k* (Number of leaves of the required subtree).

*p* (Number of physical machine clusters).

OUTPUT: T* (The approximate subtree with *k* leaves).

 1: Partition *G* into *p* clusters: {*C*_*i*_}.

 2: Sort {*C*_*i*_} in ascending order according to *w*(*C*_*i*_): *C*_1_, *C*_2_, …, *C*_*p*_.

 3: Select the first *t* clusters (*C*_1_, *C*_2_, …, *C*_*t*_), where $\Sigma_{i=1}^{t}|C_{i}\left|-t-k\in [0,\right|C_{t}\left|\right)$.

 4: **for** *i* = 0 to *t* **do**

 5:  **if** *i* ≠ *t* **then**

 6:   Create the minimum spanning tree *T*_*i*_ in *C*_*i*_ with |*C*_*i*_| − 1 leaves, the root of which is *r*_*i*_.

 7:  **else**

 8:   Create the minimum spanning tree *T*_*t*_ in *C*_*t*_ with $k-\sum_{i=1}^{t-1}\left(\right|C_{i}\left|-1\right)$ leaves, the root of which is *r*_*t*_.

 9:  **end if**

 10: **end for**

 11: Union({*T*_*i*_}) returns the approximate subtree T* with *k* leaves by joining all the roots *r*_*i*_ of *T*_*i*_ after running the classical minimum spanning tree algorithm.

## Results

### Evaluation environment and comparison schemes

The performance of the proposed cluster-and-union algorithm is demonstrated by CloudSim [34] in this section. We take the simplified intra-bandwidth consumption of DCN *φ* caused by the media server cluster, which was defined in formula 3, as the metric to evaluate the resource utilization of our scheme. The simplified intra-bandwidth consumption of DCN *φ* finally equals to the sum of the communication costs among DPs at which the media servers are located. It has the dimensions of hops. A lower *φ* value represents better resource utilization of the DCN resources. To show the effectiveness of the proposed algorithm, the simulation also sets up a comparison study. We use the straightforward random solution and the variation of the cluster-and-cut scheme [18] as the benchmarks. Different from the propose algorithm, the random solution and the cluster-and-cut scheme require knowledge of the topology structure of the media server cluster. The traffic rate matrix *D* should be known in advance. Therefore, in the comparison study, the k-leaf tree topology obtained using the proposed algorithm is utilized as the input of the two benchmarks.

In detail, the default simulation settings are as follows. Four DCN topologies, i.e., Tree, Fat-tree, VL2, and BCube, are selected. The DCN can support 1024 DPs. The media server cluster is constructed with at least 32 leaf nodes. In this manner, the bandwidth requirement of a real-time live streaming service can be quantified and guaranteed. In the experimental study, there are three scenarios. First, the number of deployment points is set to 1024, and the number of leaf nodes of the media server cluster ranges from 64 to 512, which indicates the different bandwidth requirements of a live streaming service. Second, the number of deployment points ranges from 256 to 1024, and the number of leaf nodes is set to 64, which indicates the different scales of the DCN. The last scenario is similar to the first one. The number of deployment points is set to 1024, and the number of leaf nodes of the media server cluster still ranges from 64 to 512. However, different network parameters are adopted for the Tree, VL2, and BCube topologies to study the effectiveness of the proposed algorithm under different network parameters for the given DCN structures. Finally, to reduce the randomness of the simulation and experimental results, the evaluation results presented in this paper are the average results over twenty runs.

### Results for different media server cluster scales

**Fig 3:**
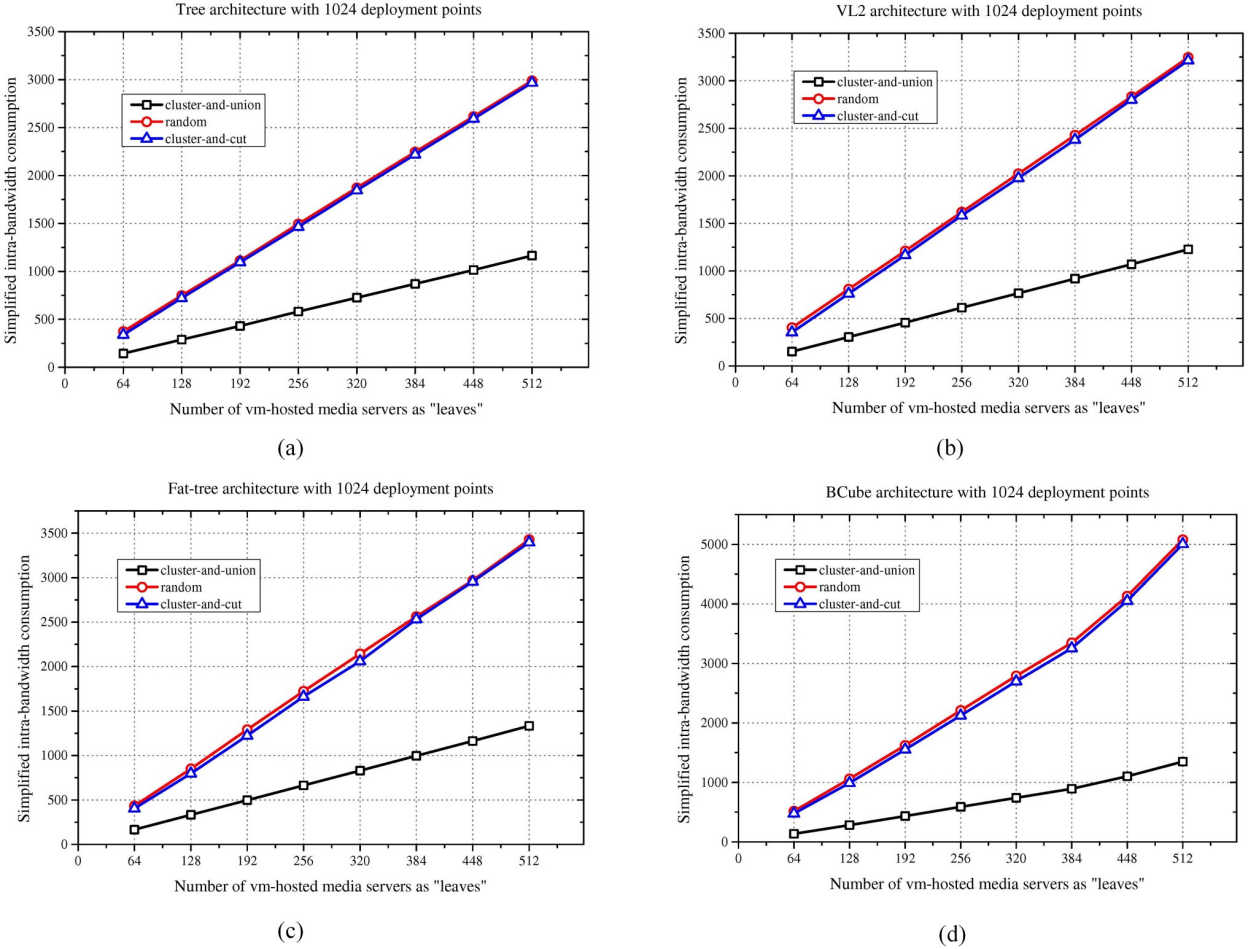
Intra-bandwidth consumption results for different scales of the media server cluster. (a) Tree. (b) VL2. (c) Fat-tree. (d) BCube.

This subsection demonstrates the efficiency of the cluster-and-union algorithm for different media server cluster scales. The simulation presents the simplified intra-bandwidth consumption results obtained using the cluster-and-union algorithm for different numbers of leaf nodes, ranging from 64 to 512, of the media server cluster. For comparison, the simulation takes the traditional random solution as a basic benchmark. At the same time, the simulation takes the cluster-and-cut scheme as an additional benchmark to show the efficiency of the proposed algorithm. The detailed results of the intra-bandwidth consumption are shown in Fig 3. With increasing scale of the media server cluster under the given 1024 DPs, compared with the traditional random or cluster-and-cut media server deployment, the cluster-and-union algorithm greatly reduces the intra-bandwidth consumption for different cluster scales. This result occurs because the proposed method clusters the media servers together to minimize the internal bandwidth consumption of the DCN. In this manner, the media server cluster is located in a local area and has a low internal bandwidth consumption. In contrast, the random method yields a random deployment result due to the uncertainty of the deployment point selection. The intra-bandwidth consumption of the DCN experiences linear growth as the scale of the media server cluster increases. It is interesting to find that the behavior of the cluster-and-cut method is the same as that of the random method. For a real-time live streaming application, the traffic rate between VM pairs is homogeneous, resulting in random clustering and cutting of the VM cluster. For this scenario, as had been indicated by the authors of the research [18], the cluster-and-cut method will provide little improvements no more than a random method. This outcome further demonstrates that the traditional traffic-aware VM management schemes in the cloud may fail to work when they do not consider the characteristics of the live streaming application.

### Results for different DCN scales

**Fig 4:**
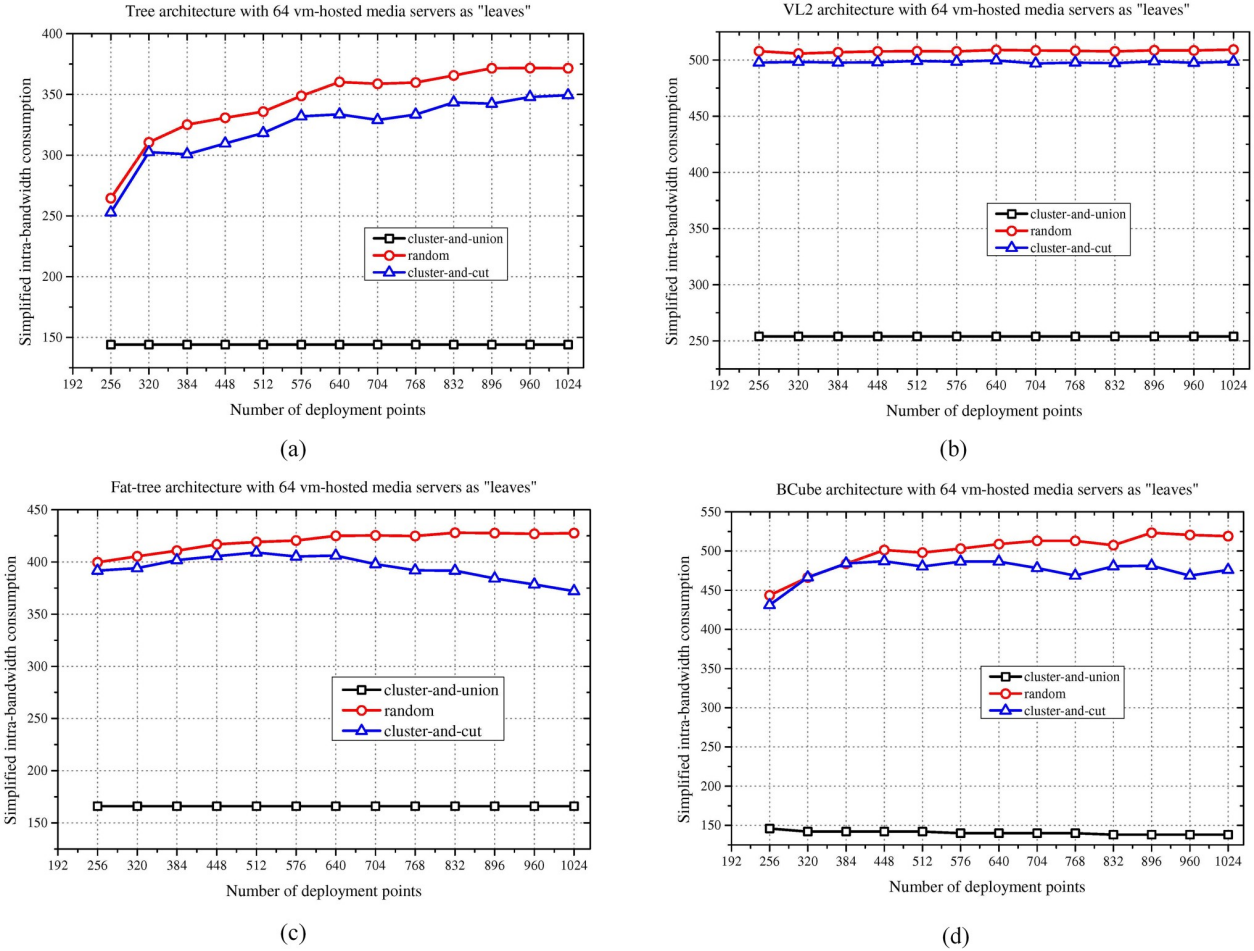
Intra-bandwidth consumption results for different DCN scales. (a) Tree. (b) VL2. (c) Fat-tree. (d) BCube.

This subsection of the simulation presents the simulation results of the cluster-and-union algorithm for different DCN scales. In this simulation, the number of leaf media server nodes is set to 64, while the scale of the DCN ranges from 256 to 1024. The detailed results regarding the simplified intra-bandwidth consumption are shown in Fig 4. From the simulation results, as the DCN scale increases under the given 64 leaf nodes of the media server cluster, the intra-bandwidth consumption essentially remains stable at a low value for our method. This outcome occurs because the proposed methods cluster together the media servers associated with the same access switches or aggregation switches. In this manner, the deployment of the media server cluster is not greatly influenced as the DCN topology scale increases. In contrast, the random method may be influenced by the DCN topology because of the uncertainty of the deployment point selection. The cluster-and-cut method and the random method behave in the same way due to the homogeneous traffic rate.

### Results for different parameters for the same DCN scale

**Fig 5:**
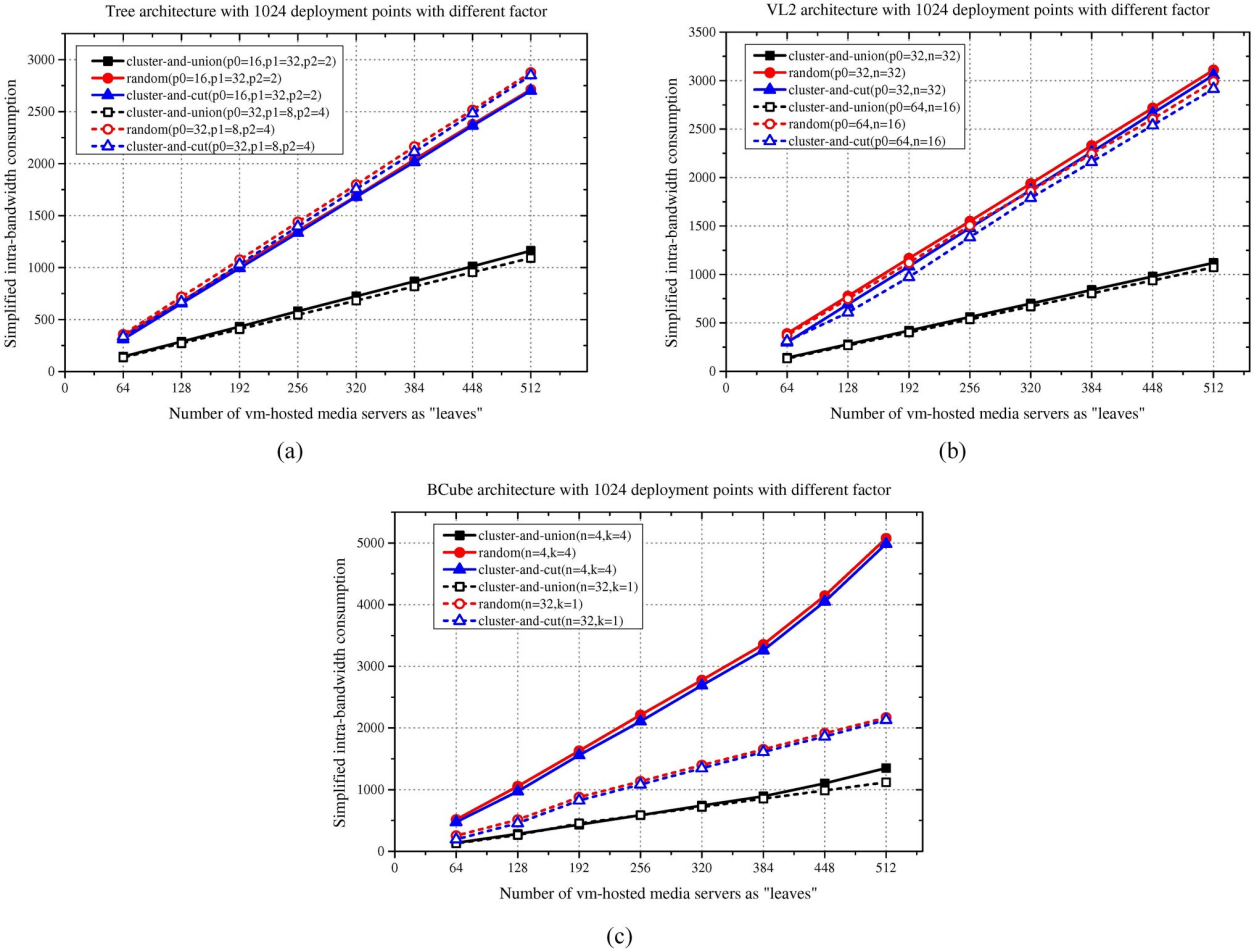
Intra-bandwidth consumption results for different parameters. (a) Tree. (b) VL2. (c) BCube.

This subsection of the simulation presents the simulation results for DCNs with the same size but different parameters. For the DCN with the Tree structure, the size of the DCN is determined as follows: *size* = *p*_0_*p_1_*p_2_, where *p*_*i*_ indicates the downward connections of the *ith* layer switches. For VL2, the size of the DCN is determined as follows: *size* = *p*_0_*n, where *p*_0_ indicates the downward connections of the access layer switches and *n* represents the number of upper layer switches. For BCube, the size of the DCN is determined as follows: *size* = *n*^(*k*+1)^, where *k* indicates the layers involved and *n* indicates the downward connections of the switches. Finally, for Fat-tree, the size of the DCN is determined as follows: *size* = *k*^3^/4, where *k* indicates the downward connections between the access layer switches and the deployment points. Since the size of the Fat-tree topology is determined by only the connections *k* between the switches and the host servers, we cannot maintain the size of the DCN while changing the value of *k*. Therefore, in this subsection, we only provide the simulation results corresponding to the Tree, VL2, and BCube topologies. In the simulation, the number of deployment points is firmly set to 1024, but the network parameters for the given DCN structures are different. In the simulation, the number of leaf nodes still ranges from 64 to 512. According to the simulation results in Fig 5, the variation of parameters has very little impact on the final results for the Tree and VL2 DCNs. The proposed scheme outperforms the benchmarks significantly. Meanwhile, the variation of parameters in BCube influences the results obtained using the random and cluster-and-cut methods. However, our proposed algorithm still stably outperforms the two benchmarks.

## Conclusion

This study addresses the VM-hosted media server cluster deployment issue that occurs in a media cloud for real-time live streaming services. An external-bandwidth-guaranteed VM placement scheme is presented to optimize the internal bandwidth utilization in a DCN as well as to guarantee the external bandwidth of a real-time live streaming application. The scheme models the optimal resource utilization problem as a terminal-Steiner-tree-like problem and demonstrates the difficulty of the problem. Furthermore, a greedy algorithm, which includes a greedy media server placement algorithm to find the minimum tree with the necessary leaf nodes, is introduced to solve the target problem. From the evaluation results, when compared with the traditional methods for different DCN topologies and media server cluster settings, the proposed scheme achieves a lower DCN intra-bandwidth consumption under the ultra-bandwidth constraint, which is beneficial for enlarging the application scale while still guaranteeing a satisfactory external bandwidth for the service.

In this study, although the proposed scheme considers the external bandwidth requirement of a real-time live streaming application, some other quality of service parameters or quality of experience metrics of live streaming application are not addressed. For example, the future work should include of designing a quality of experience driven VM placement method for live streaming services. Furthermore, future work should also attempt to devise a dynamic media server migration method in accordance with the dynamic nature of the cloud. Finally, in addition to an evaluation via simulation, future work should evaluate the efficiency of the algorithm in a real cloud-based virtual classroom environment and obtain experimental judgments.

## Supporting information

S1 Dataset Original data of experiments. (ZIP)
